# Supplementary material for: Preventive Effects of the Marine Microalga Phaeodactylum tricornutum, Used as a Food Supplement, on Risk Factors Associated with Metabolic Syndrome in Wistar Rats
Source: Nutrients. 2019 May 14;11(5):1069. doi: 10.3390/nu11051069 (PMC6566425; doi:10.3390/nu11051069)
Supplement: Supplementary file 1 [file nutrients-11-01069-s001.pdf]

Supplementary materiel

Table S1. Biochemical composition of standard, high-fat diets and freeze-dried *P. tricornutum* biomass

	**CTRL**	**HF**	**Phaeo**	
Nutrients (g/100g)	Data*	Data*	Mean	SD
Proteins	16	19	38.90	0.85
Carbohydrates	60	32	22.60	0.41
Lipids	4	36	15.80	
Mineral and vitamin mixture	5	5	-	
TDF†	4	-	11.17	
IDF‡	-	-	6.29	
SDF‡	-	-	6.06	
Humidity	11	8	2.43	0.16
Ash	-	-	23.60	0.15

CTRL, standard diet; HF, high-fat diet; Phaeo, freeze-dried of *P. tricornutum*; IDF, insoluble dietary fiber; TDF, total dietary fiber; SDF, soluble dietary fiber; -, not analysed. For CTRL diet, the mineral mixture provides the following amounts in mg/kg of diet: C, 7300; Cl, 4000; Cu, 16; Fe, 270; K, 6000; Mg, 1600; Mn, 70; Na, 2500; P, 5500; Zn, 55. The vitamin mixture of CTRL diet provides the following amounts in mg/kg of diet: biotin, 0.04; cholin, 1600; folic acid, 0.5; niacin, 75; vitamin A, 7500; vitamin B1, 7; vitamin B2, 6.5; vitamin B5, 16.5; vitamin B6, 2.6; vitamin B12, 0.02; vitamin D3, 1000; vitamin E, 30; vitamin K3, 2.5. For HF diet the mineral mixture provides the following amounts in mg/kg of diet: Ca, 3000; Na, 4100; P, 5000. Results are represented as mean values ± SD, $n = 3$ for proteins, carbohydrates, humidity and ash of Phaeo biomass. * Data of biochemical composition of control and high-fat diets have been furnished by SAFE; † Mean values ($n = 2$) are shown for TDF of Phaeo biomass. ‡ Values ($n = 1$) are shown for IDF and SDF of Phaeo biomass.

Table S2. Fatty acid composition of diets

	CTRL (mol %)	HF (mol %)	Phaeo (g/100g)	
Fatty acid	Mean	Mean	Mean	SD
4:0	-	1.49	ND	ND
8:0	-	1.20	ND	ND
10:0	-	3.07	ND	ND
12:0	-	3.95	ND	ND
14:0	0.63	13.16	0.64	0.02
14:1	-	-	0.03	0.05 10^{-2}
15:0	-	-	0.02	0.01 10^{-1}
16:0	15.91	32.87	1.37	0.04
16:1n-7	0.73	-	1.27	0.06
16:2n-6	-	-	0.09	0.09 10^{-1}
16:3n-3	-	-	0.64	0.03
16:3n-6	-	-	0.22	0.00
16:4n-1	-	-	0.01	0.01 10^{-1}
16:4n-6	-	-	0.04	0.00
18:0	2.00	13.32	0.19	0.02 10^{-1}
18:1n-7	-	-	0.20	0.03 10^{-1}
18:1n-9	21.44	16.92	0.34	0.11
18:2n-6	49.84	5.44	0.19	0.06 10^{-1}
18:3n-3	4.23	-	0.06	0.01 10^{-1}
18:3n-6	-	-	0.04	0.03 10^{-1}
18:4n-3	1.28	-	0.05	0.07 10^{-1}
20:0	-	-	0.03	0.03 10^{-2}
20:2n-6	-	-	0.02	0.01 10^{-1}
20:3n-3	-	-	0.03	0.08 10^{-2}
20:4n-3	-	-	0.04	0.03 10^{-1}
20:4 n-6	-	-	0.15	0.09 10^{-1}
20:5 n-3	0.66	-	2.04	0.15
22:0	-	-	0.07	0.01 10^{-1}
22:1	-	-	0.04	0.02 10^{-2}
22:5n-3	0.91	-	ND	ND
22:5n-6	-	-	0.04	0.02 10^{-2}
22:6n-3	1.03	-	0.25	0.05 10^{-1}
24:0	-	-	0.26	0.08 10^{-1}
Others	1.24	8.58	0.09	0.00
Total SFA	18.54	69.06	2.58	0.07
Total MUFA	22.17	16.92	1.88	0.06
Total PUFA	58.05	5.44	3.91	0.18
Total n-3 PUFA	8.11	-	3.11	0.12
Total n-6 PUFA	49.94	5.46	0.79	0.02
n-6 PUFA/ n-3 PUFA	0.16	-	0.25	0.01
Total fatty acids	98.76	91.42	8.37	0.13

CTRL, standard diet; HF, high-fat diet; ND, not detected; Phaeo, freeze-dried of *P. tricornutum*; -, not analysed. Results are represented as mean values ± SD, n = 3 for Phaeo biomass. Mean values from CTRL and HF diets (n = 2) were similar, no statistical analysis were carried out, no SD mentioned.

Table S3. Pigment and sterol composition, antioxidant activity and *in vitro* digestibility of freeze-dried *P. tricornutum*

	**Phaeo**	
Pigments (g/100g)	Mean	SD
Total chlorophyll	1.49	0.05 10^{-1}
Chlorophyll-*a*	1.16	0.02
Chlorophyll-*c*	0.33	0.03 10^{-1}
Total carotenoids	0.55	0.01
Carotenoids (g/100g)		
Fucoxanthin	0.31	0.06 10^{-1}
4k-hex-fucoxanthin	0.12	0.04
Diadinoxanthin	0.03	0.02 10^{-1}
Diatoxanthin	0.95 10^{-2}	0.05 10^{-2}
β-carotene	0.26 10^{-1}	0.05 10^{-2}
Others	0.52 10^{-1}	0.03 10^{-1}
Sterols (g/100g)		
Cholesterol	6.50 10^{-3}	
Brassicasterol	5.94 10^{-1}	
24-Metilcholesterol	3.25 10^{-3}	
Campesterol	2.93 10^{-2}	
β-sitosterol	1.11 10^{-2}	
Sitostanol	1.05 10^{-3}	
Others	4.55 10^{-3}	
Total sterols	0.65	
Antioxidant activity*		
Antiradical activity (I_{50})† (DPPH in mg biomass/ml extract)‡	1.81	0.15
Antioxydant activity ORAC (μmol TE/mg biomass)§	106.39	6.92
In vitro digestibility (% dry matter)	52.00	2.90

DPPH, 2, 2-diphenyl-1-picrylhydrazyl; ORAC, Oxygen-radical absorbance capacity; Phaeo,freeze-dried of *P. tricornutum*; TE, Trolox equivalent. Results are represented as mean values ± SD, *n* = 3, excepted for sterol composition (*n* = 1). * Antioxidant activity was measured from extracts of Phaeo in 90% acetone. † I_{50}: concentration of the biomass/mL extract decreasing the absorbance of the DPPH solution by 50%. ‡ DPPH is a stable radical that can be reduced by reaction with an antiradical hydrogen-donor compound. § Trolox: water-soluble vitamin E analogue. 1 ORAC unit equals the net protection produced by 1 μM Trolox.

Table S4. Total fatty acid composition of red blood cells

	CTRL		HF		HF-Phaeo	
Fatty acid (mol %)	Mean	SD	Mean	SD	Mean	SD
**SFA**						
14:0	0.73	0.16	1.00	0.32	1.01	0.14
16:0	37.91	1.93	27.93***	2.62	32.72***†	3.79
18:0	13.18	0.99	13.48	1.08	14.40	1.06
Total SFA	51.82	1.93	42.40**	3.93	48.13	4.93
**MUFA**						
16:1	0.98	0.12	0.97	0.12	0.96	0.17
18:1(n-7 + n-9)	11.38	0.79	13.31*	1.41	11.43	0.51
20:1	ND	-	ND	-	ND	-
22:1	0.57	0.10	0.95***	0.25	0.23***†	0.09
24:1n-9	ND		ND		ND	
Total MUFA	12.93	0.86	15.22**	1.26	12.62†	0.51
**PUFA**						
18:2n-6	6.79	0.66	7.78	0.77	6.08†	0.95
20:2n-6	0.31	0.05	0.82**	0.14	0.49†	0.18
20:4n-6	10.33	0.87	15.80***	2.24	8.87†	3.00
n-6 PUFA	17.17	1.65	24.01***	2.38	13.95†	2.28
18:3n-3	ND	-	ND	-	ND	-
20:5n-3	0.52	0.18	0.30	0.09	1.77***†	0.42
22:5n-3	10.68	2.09	7.11	1.73	14.32†	3.87
22:6n-3	1.23	0.19	2.43***	0.28	1.51†	0.51
n-3 PUFA	12.99	2.06	9.78	1.90	18.54**†	3.57
**n-6 PUFA/n-3 PUFA**	1.41	0.27	2.41**	0.36	1.12†	0.13
**Total PUFA**	30.08	2.02	32.86	3.06	32.68	5.82
**MUFA/SFA**	0.25	0.02	0.36***	0.02	0.26†	0.02
**Δ9-Desaturase index‡**	0.46	0.03	0.50	0.02	0.44†	0.01

CTRL, standard diet; HF, high-fat diet; HF-Phaeo, high-fat diet supplemented with 12% of *P. tricornutum*; MUFA, mono-unsaturated fatty acids; ND, not detected; PUFA, polyunsaturated fatty acids; SFA, saturated fatty acids. Results are represented as mean values ± SD, n = 6. Mean values were significantly different from those of the CTRL group: *$p < 0.05$; **$p < 0.001$; ***$p < 0.0001$ (one-way ANOVA; LSD post hoc test). † significant difference compared with the HF group ($p < 0.05$). ‡ Δ9-Desaturase index = [18:1(n-7 + n-9)/ 18:0 + 18:1(n-7 + n-9)].

Table S5. Fatty acid composition of liver total lipids

	**CTRL**		**HF**		**HF-Phaeo**	
Fatty acid (mol %)	Mean	SD	Mean	SD	Mean	SD
**SFA**						
14:0	0.31	0.08	1.80***	0.10	0.95***†	0.11
16:0	20.85	0.98	22.23	3.06	20.40	0.89
18:0	13.52	0.73	13.00	2.37	15.48†	1.71
Total SFA	34.54	0.31	37.03	4.35	36.84	1.65
**MUFA**						
16:1	2.13	0.46	2.90	0.98	1.86†	0.51
18:1(n-7 + n-9)	13.18	1.65	17.77	7.15	16.08	1.27
20:1n-9	ND	-	ND	-	ND	-
22:1	0.44	0.09	0.26***	0.09	0.17***	0.02
24:1n-9	0.41	0.05	3.09***	0.26	1.29***†	0.23
Total MUFA	17.38	1.70	24.01	8.02	20.15	1.09
**PUFA**						
18:2n-6	15.81	1.89	9.58***	1.45	9.44***	0.82
20:2n-6	0.47	0.18	0.59	0.46	0.22	0.06
20:4n-6	20.83	1.58	9.35***	1.88	15.49***†	1.01
n-6 PUFA	34.09	2.21	19.52***	0.52	25.15***†	1.43
18:3n-3	0.40	0.09	0.44	0.12	0.25*†	0.04
20:5n-3	0.72	0.08	0.25***	0.08	1.73***†	0.17
22:5n-3	0.91	0.10	0.72	0.13	2.40***†	0.21
22:6n-3	7.09	1.23	3.58***	0.87	7.65†	0.72
n-3 PUFA	8.00	1.22	5.10	0.77	12.41**†	0.82
**n-6 PUFA/n-3 PUFA**	4.34	0.79	3.88	0.51	2.04***†	0.25
**Total PUFA**	45.86	1.69	24.61***	1.29	37.56***†	0.74
**MUFA/SFA**	0.51	0.06	0.67	0.29	0.53	0.04
**Δ9-Desaturase index‡**	0.49	0.04	0.56	0.14	0.51	0.02

CTRL, standard diet; HF, high-fat diet; HF-Phaeo, high-fat diet supplemented with 12% of *P. tricornutum*; MUFA, mono-unsaturated fatty acids; ND, not detected; PUFA, polyunsaturated fatty acids; SFA, saturated fatty acids. Results are represented as mean values ± SD, $n = 6$. Mean values were significantly different from those of the CTRL group: *$p < 0.05$; **$p < 0.001$; *** $p < 0.0001$ (one-way ANOVA; LSD post hoc test). † significant difference compared with the HF group ($p < 0.05$). ‡ Δ9-Desaturase index = [18:1(n-7 + n-9)/ 18:0 + 18:1(n-7 + n-9)].

Table S6. Fatty acid composition of liver neutral lipids

	CTRL		HF		HF-Phaeo	
Fatty acid (mol %)	Mean	SD	Mean	SD	Mean	SD
**SFA**						
14:0	0.66	0.15	2.17***	0.30	1.79***†	0.18
16:0	23.57	1.55	26.92*	1.93	24.86	2.34
18:0	1.67	0.67	4.83**	0.58	2.76†	0.64
Total SFA	25.25	1.94	35.11**	2.94	30.58**†	2.15
**MUFA**						
16:1	4.30	0.79	4.31	0.41	3.40	0.67
18:1(n-7 + n-9)	25.25	2.56	36.24***	3.31	27.85†	3.76
20:1n-9	0.30	0.13	0.44	0.17	0.11†	0.01
22:1	0.25	0.05	0.13**	0.03	0.16**	0.01
24:1n-9	2.00	0.84	1.47	0.66	4.89***†	1.25
Total MUFA	31.82	1.64	42.70**	3.73	38.05**	1.19
**PUFA**						
18:2n-6	23.78	2.49	8.84***	1.25	9.05***	0.62
20:2n-6	0.35	0.10	0.25	0.08	0.17**	0.03
20:4n-6	5.49	1.16	3.01**	1.40	1.61**	0.21
n-6 PUFA	29.62	2.74	11.30***	1.53	10.74***	0.43
18:3n-3	1.05	0.16	0.45***	0.13	0.53***	0.09
20:5n-3	0.72	0.08	0.25***	0.08	1.73***†	0.17
22:5n-3	0.85	0.24	0.44***	0.11	2.32***†	0.35
22:6n-3	3.02	0.34	0.90***	0.19	3.02†	0.68
n-3 PUFA	5.64	0.36	2.08***	0.39	10.74***†	0.89
**n-6 PUFA/n-3 PUFA**	5.26	0.41	5.10	0.41	1.68***†	0.18
**Total PUFA**	35.25	2.95	12.49***	1.23	16.59***†	2.32
**MUFA/SFA**	1.12	0.27	1.16	0.14	1.25	0.08
**Δ9-Desaturase index‡**	0.92	0.03	0.86*	0.04	0.92†	0.02

CTRL, standard diet; HF, high-fat diet; HF-Phaeo, high-fat diet supplemented with 12% of *P. tricornutum*; MUFA, mono-unsaturated fatty acids; PUFA, polyunsaturated fatty acids; SFA, saturated fatty acids; . Results are represented as mean values ± SD, $n = 6$. Mean values were significantly different from those of the CTRL group: *$p < 0.05$; **$p < 0.001$; ***$p < 0.0001$ (one-way ANOVA; LSD post hoc test). † significant difference compared with the HF group ($p < 0.05$). ‡ Δ9-Desaturase index = [18:1(n-7 + n-9)/ 18:0 + 18:1(n-7 + n-9)].

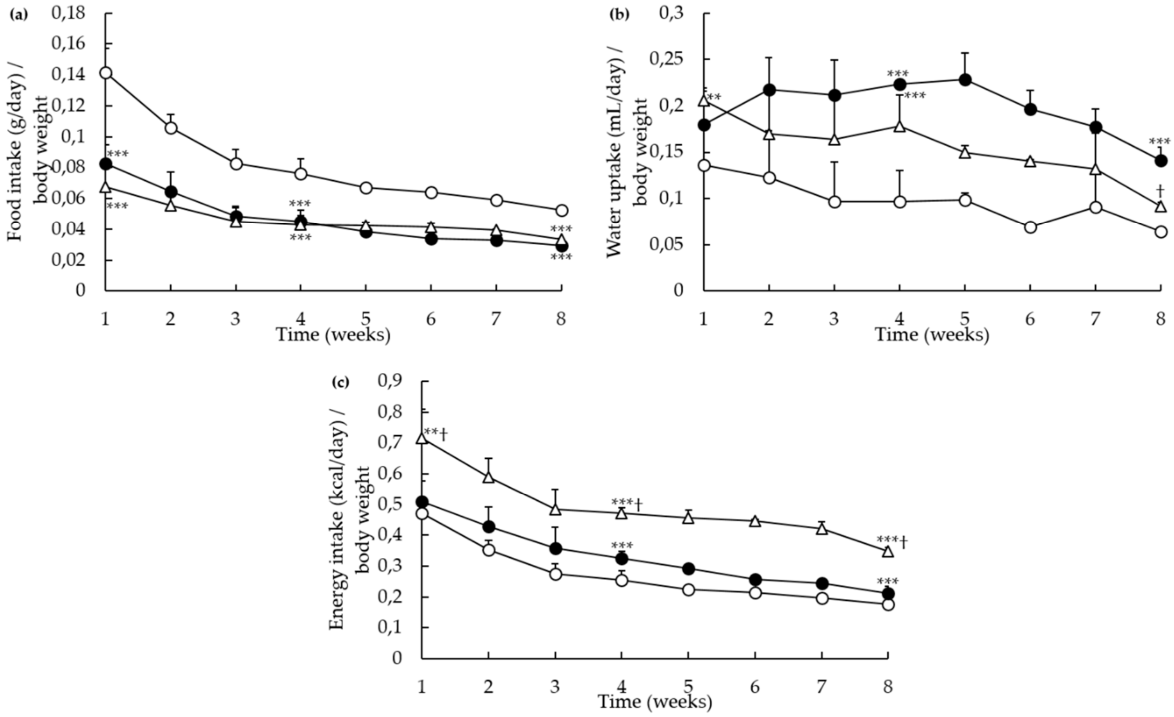

Figure S1. Comparaison of daily food intake **(a)**, water intake **(b)**, energy intake **(c)** between the different groups. CTRL (─○─), control group; HF (─●─), high fat group; HF-Phaeo (─△─), high fat group supplemented with *P. tricornutum*. Values are means (n = 6), with standard deviations represented by vertical bars. Mean values were significantly different from those of the CTRL group: *$p < 0{\cdot}05$; **$p < 0{\cdot}001$; ***$p < 0{\cdot}0001$ (one-way ANOVA; LSD post hoc test). † significant difference compared with the HF group ($p < 0{\cdot}05$).
